# Influence of Manipulating Running Foot Strike Angle on Internal Loading of the Tibia

**DOI:** 10.1111/sms.70066

**Published:** 2025-05-11

**Authors:** Sanghyuk Han, Jeheon Moon, Jusung Lee, Sooji Han, Thorsten Sterzing, Dominic Farris, Hannah Rice

**Affiliations:** ^1^ Public Health and Sport Sciences University of Exeter Exeter UK; ^2^ Human Performance Laboratory Descente Innovation Studio Complex Busan Republic of Korea; ^3^ Department of Physical Education Korea National University of Education Cheongju‐si Republic of Korea; ^4^ University Industry Foundation Pukyong National University Busan Republic of Korea; ^5^ Department of Physical Performance Norwegian School of Sport Sciences Oslo Norway

**Keywords:** beam theory, foot strike pattern, internal tibial loading, musculoskeletal modeling, running injury

## Abstract

Tibial stress injuries are problematic among runners. Foot strike pattern upon landing may alter internal tibial loading, which could potentially affect the risk of stress injuries during running. The purpose of this study was to quantify internal loading at the distal 1/3 of the tibia during running with imposed rearfoot and forefoot strikes. Nineteen habitual rearfoot strikers were recruited to run with their preferred foot strike and then with imposed rearfoot and forefoot strikes in a randomly assigned order. Force and motion capture data were collected synchronously, and the foot strike in the sagittal plane was identified from the kinematic data. The tibial bending moments were estimated using musculoskeletal modeling and beam theory, and cumulative‐weighted tibial impulse per kilometer was derived. Significant differences in peak bending moments were found among foot strike patterns (*p <* 0.001). Running with an imposed forefoot strike increased tibial loading, especially during early to mid‐stance (2%–67% stance, *p <* 0.001). However, imposed rearfoot striking resulted in lower bending moments than both habitual rearfoot striking (*p <* 0.001) and forefoot striking (*p <* 0.001). Additionally, cumulative‐weighted impulse per kilometer was significantly greater when running with an imposed forefoot strike compared to both habitual (*p* = 0.001) and imposed rearfoot strikes (*p <* 0.001). Running with an imposed nonhabitual forefoot strike results in higher tibial loading than rearfoot striking due to increased mechanical demands placed on the plantar flexors. Transitioning from a habitual rearfoot strike to a forefoot strike may not be advisable for runners aiming to reduce tibial loading.

## 1 Introduction

Running is a popular sport worldwide that is associated with a relatively high risk of lower limb injuries, reportedly in the range from 20% to 79% [1]. Lower limb stress injuries are a problematic injury among runners [2], especially in the tibia, which is one of the most common sites of injury [3]. Tibial stress injuries develop from the accumulation of microdamage to the bone caused by repetitive loading [4, 5]. Although appropriate bone loading can be beneficial [6], excessive loading can lead to the accumulation of fatigue damage, which may increase the risk of stress fractures [7]. Quantifying tibial loading directly during human movement requires invasive in vivo measurements. Lanyon et al. [8] were the first to use strain gauges attached to the distal anteromedial tibia of a male participant during running, providing valuable insights into bone deformation under load. Additional studies have been conducted to quantify the internal strain at the anteromedial tibia during running, with measurements ranging between 1000 and 2000 *με* [9, 10, 11]. However, these studies were limited by small sample sizes and the restricted location at which strain gauges could be attached. This method also requires costly surgical procedures and its invasive nature may alter how people run compared to real‐world settings.

Given these limitations, computational approaches have been developed to estimate in vivo loadings. Musculoskeletal modeling and beam theory methods have been employed to quantify tibial loads during running [12, 13, 14, 15]. Although estimates of tibial loading using beam theory tend to underestimate values compared to the more computationally expensive finite element approaches, the values still exhibit a high correlation with finite element model estimates [12], suggesting they are suitable for within‐participant study designs. Rice et al. [14] also observed that this approach is repeatable when quantifying peak anterior and posterior tibial stresses during running.

The interaction between loading magnitude and frequency is crucial for evaluating the risk of stress injuries. Cumulative loading metrics consider both factors, but proper weighting of magnitude is necessary to avoid overemphasizing frequency. Foot strike patterns could influence stride frequency, loading magnitude, and ground contact time, and these effects can be quantified using a weighted impulse, as shown in previous studies [16, 17, 18]. This approach may help to understand the mechanisms of tibial stress injury associated with foot strike patterns.

Running foot strike patterns have been identified as modifiable factors that can affect impact kinetics [19]. Daoud et al. [19] conducted a retrospective cohort study and observed a lower rate of overuse injuries in forefoot strikers compared to rearfoot strikers, although these findings did not specifically address internal tibial loading. Similarly, the transition from rearfoot striking to forefoot striking has been associated with reduced peak and average loading rates during running [20], suggesting a potential link to bone stress injury [21]. Chen et al. [22] used a probabilistic model to evaluate how foot strike patterns influence tibial stress fracture risk. Their findings indicated that while forefoot striking reduced vertical loading rates, it did not significantly alter peak tibial strains or the risk of stress fracture. This highlights the importance of analyzing internal tibial loads, as ground reaction forces may not reliably reflect internal loading [23]. Edwards [16] also noted that the musculoskeletal system may not be mechanically affected by higher loading rates, as biological materials are often more resilient to short‐duration mechanical loads. These insights highlight the limitations of using external loading variables alone to assess overuse injuries in musculoskeletal structures [24].

Recent studies have explored the effects of foot strike patterns and other gait modifications on tibial loading and injury risk. For instance, Huang et al. [25] investigated the combined effects of foot strike pattern, step rate, and anterior trunk lean on impact loading, concluding that a forefoot strike combined with an increased step rate led to the lowest impact loading rates. Similarly, Yong et al. [26] demonstrated that converting to a forefoot strike reduced average and peak loading rates associated with tibial stress fracture risk. However, these studies primarily focused on external metrics such as ground reaction forces and tibial acceleration, which may not represent the internal tibial loads that directly contribute to injury mechanisms. Quantifying internal loading may provide a better understanding of the influence of different foot strike patterns on tibial loading than these surrogate measures.

According to a systematic review, the influence of transitioning to forefoot striking on tibial loading remains inconclusive [27]. Given that the work done at the ankle is increased when running with a forefoot strike [28], which may in turn increase tibial loads via increased muscular‐tendinous forces, it is important to quantify internal loading. Numerous studies have recruited habitual rearfoot strikers and asked them to adopt a forefoot strike, which likely results in them exaggerating this foot strike [20]. When asking habitual rearfoot runners to ensure they run with an imposed rearfoot strike, this same exaggeration effect may be observed. The purpose of this study was to identify the influence of running with an imposed rearfoot strike and forefoot strike on estimated tibial loading compared with a habitual rearfoot strike. It was hypothesized that running with a forefoot strike would increase internal loading of the tibia compared with running with a habitual or imposed rearfoot strike.

## 2 Methods

### 2.1 Participants

Nineteen healthy recreational runners (10 females, 9 males; age 34.0 *±* 4.8 years; height: 166.4 *±* 6.3 cm; weight: 61.6 *±* 6.6 kg; mean *±* SD) were recruited from local running clubs to participate in this study. All participants self‐reported a habitual rearfoot strike during running, which was verified using foot strike angle (FSA) at initial contact defined as the angle between the foot and the ground in the sagittal plane. A rearfoot strike was classified as having an FSA greater than 8°, following previously established thresholds [29]. Participants were excluded if they had a history of any lower limb musculoskeletal injuries in the past year or if they did not habitually run with a rearfoot strike. All the participants provided written informed consent prior to participation according to a protocol approved by the Korea Institute of Sport Science Ethics Committee (KISS‐1907‐018‐01).

### 2.2 Experimental Protocols

Participants were provided with athletic clothing and wore their own running shoes during the experiment. A total of 50 retro‐reflective markers were positioned to identify the anatomical frames of the thorax, pelvis, upper arm, lower arm, thigh, shank, and foot. Marker coordinates were tracked at a sampling frequency of 200 Hz using 18 infrared cameras, in conjunction with 3D motion capture software (Oqus 7+; Qualisys, Göteborg, Sweden). Ground reaction forces were recorded at a sampling frequency of 2000 Hz using a force plate (9287BA; Kistler, Winterthur, Switzerland), synchronized with the motion capture system.

**FIGURE 1:**
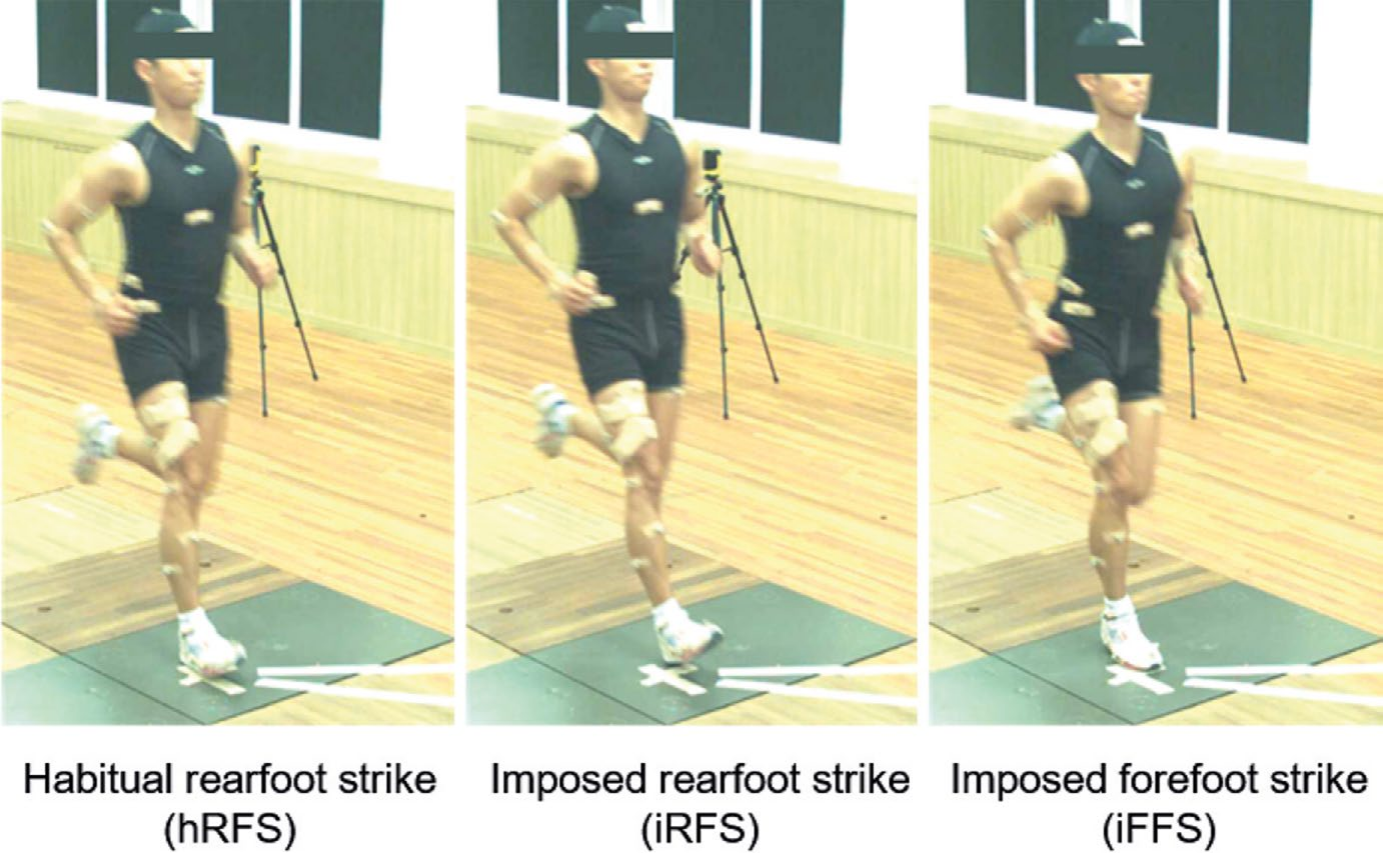
Participants' foot strike patterns from habitual rearfoot strike (hRFS) to imposed rearfoot strike (iRFS) and imposed forefoot strike (iFFS).

Participants were given time to complete a self‐directed warm‐up. They were then asked to run overground at 4.0 m/s along a 10 m runway, using their habitual foot strike, ensuring a full right foot contact on the force plate. The running speed (*±*5%) was verified using timing gates (Witty, Microgate, BZ, Italy) with two sets of gates installed at 2.5 m intervals centered around the force plate. After five successful trials, participants were instructed to modify their foot strike patterns from a habitual rearfoot strike to an imposed rearfoot strike or an imposed forefoot strike (Figure 1). The order of running with an imposed rearfoot strike or an imposed forefoot strike was randomly assigned, and the modified foot strike patterns were performed at the same speed. Five successful trials were collected for each foot strike pattern. Participants were given specific instructions to familiarize themselves with each imposed foot strike pattern, with a dedicated familiarization session before each set of trials. For each condition, participants completed at least 10 familiarization trials specific to the assigned foot strike pattern before beginning the actual data collection. This pattern was repeated for each foot strike condition (i.e., familiarization‐practice followed by actual trials for each condition). Participants were encouraged to run naturally throughout the trials and were not instructed to specifically aim for the force plate. They were allowed to practice as needed before each trial to ensure comfort with the assigned foot strike pattern. During the experimental trials, one investigator visually monitored the foot strike pattern, and motion capture data were reviewed if the pattern appeared ambiguous or incorrect. Participants received corrective verbal feedback when needed to help them adopt the assigned foot strike pattern.

### 2.3 Data Processing

Marker coordinates and force data were processed using a low‐pass fourth‐order zero‐lag Butterworth filter, at cutoff frequencies of 10 and 20 Hz, respectively. Kinematic and kinetic data were analyzed using Visual3D software (V6; HAS‐Motion, Ontario, Canada) for the stance phase of running. The stance phase was defined as the time period during which the filtered vertical ground reaction force exceeded 10 N. Data were time‐normalized to 101 points (0%–100% stance). Static trials were used to determine the joint center locations of the ankle, knee, and hip for each participant on both sides. Joint angles were computed using the XYZ Cardan sequence, where flexion–extension was represented by the *X* axis, abduction–adduction by the *Y* axis, and internal–external rotation by the *Z* axis. Each joint was modeled with 6 degrees of freedom, accounting for three rotational and three translational movements. For net joint moments, the right‐hand rule was applied to determine the direction of rotation, with positive values representing counterclockwise rotation in the sagittal plane. Joint reaction forces were calculated using inverse dynamics within a customized MATLAB (R2021a; MathWorks, Natick, MA, USA) script, based on each participant's anthropometric measurements [30], which included mass, height, and gender. These anthropometric measurements were used to calculate the center of mass and inertial properties of the segments.

To estimate tibial bending moments, a customized MATLAB script was employed, as used previously [15]. Dynamic muscular forces were estimated through static optimization, using the fmincon function in MATLAB, which minimized the sum of cubed muscle stresses [31]. Using this function, the forces were constrained to be equal to the joint moments in the sagittal plane. The bending moments about the medial–lateral axis—quantified in the present study—have previously been shown to be robust to the choice of joint moment constraint [32]. Eleven muscles spanning the distal third of the tibia were included in the model: lateral gastrocnemius, medial gastrocnemius, tibialis anterior, soleus, tibialis posterior, extensor digitorum longus, flexor digitorum longus, flexor hallucis longus, peroneus brevis, peroneus longus, and extensor hallucis longus. The physiological cross‐sectional areas of these muscles were used to calculate the maximum isometric muscular forces, assuming a specific tension of 61 N/cm^2^ [33]. Moment arms for each muscle, along with the muscle‐tendon coordinates including muscle origins, insertions, and wrapping points, were obtained from the Hamner model [34].

The resultant bending moment $M_{\text{resultant}}$ at 33% of the distance from the distal end of the tibia, which corresponds to the narrowest cross‐sectional area [35], was estimated as the sum of the muscular bending moment $M_{\text{muscle}}$ and the joint reaction bending moment $M_{\text{reaction}}$, as shown in Equation (1):

(1) $$M_{\text{resultant}}=M_{\text{muscle}}+M_{\text{reaction}}$$

The muscular component of the tibial bending moment is described in Equation (2):

(2) $$M_{\text{muscle}}=\sum_{i=1}^{11}F_{m_{i}}\cdot \sin\theta_{i}\cdot \left(L_{\text{tibia}}-L_{67\%\text{prox}}\right)$$

The joint reaction force component of the tibial bending moment is provided in Equation (3):

(3) $$M_{\text{reaction}}=F_{\mathrm{jrf}}\cdot \sin\beta \cdot \left(L_{\text{tibia}}-L_{67\%\text{prox}}\right)$$

where $F_{m_{i}}$ is the force generated by the *i*th muscle, $\theta_{i}$ is the sagittal plane angle between the longitudinal axis of the tibia and the vector of the *i*th muscular force, and $L_{\text{tibia}}$ is defined as the total length of the tibia. $L_{67\%\text{prox}}$ refers to the length measured from the proximal end of the tibia to the point of interest. $F_{\mathrm{jrf}}$ is the external joint reaction force acting at the ankle, and β is the sagittal angle between the longitudinal axis of the tibia and the resultant joint reaction force vector. The muscular component and joint reaction force components of the tibial bending moment are henceforth referred to as the muscular component and JRF component, respectively.

Cumulative‐weighted tibial impulse was calculated using the method proposed by Firminger et al. [17], which incorporates a tissue‐dependent weighting factor to estimate loading over a kilometer of running, as shown in Equation (4):

(4) $$\text{Cumulative}-\text{weighted tibial impulse}=n{\left[\int_{t_{i}}^{t_{f}}{\left(x_{s}\right)}^{b}\mathrm{dt}\right]}^{\frac{1}{b}}$$

where n represents the number of right foot contacts, $t_{i}$ and $t_{f}$ denote the start and end times of the stance phase, and $x_{s}$ is the time‐series data of internal tibial bending moment. The parameter b is a tissue‐dependent weighting factor that accounts for the nonlinear relationship between applied stress/strain and the useful life of fatigue. For bone, b is set to 6.6 based on experimental data from Carter et al. [36]. The weighted impulse approach accounts for the fact that the magnitude of the loading is more critical to the cumulative tissue strain than the number of loading cycles [16, 37]. The number of steps was calculated using the average step frequency, derived as the inverse of the stride duration (1/stride duration), for each kilometer. This calculation represents the total number of contacts per kilometer of the right foot, as the analysis focuses only on the cumulative strain experienced by the right tibia.

**FIGURE 2:**
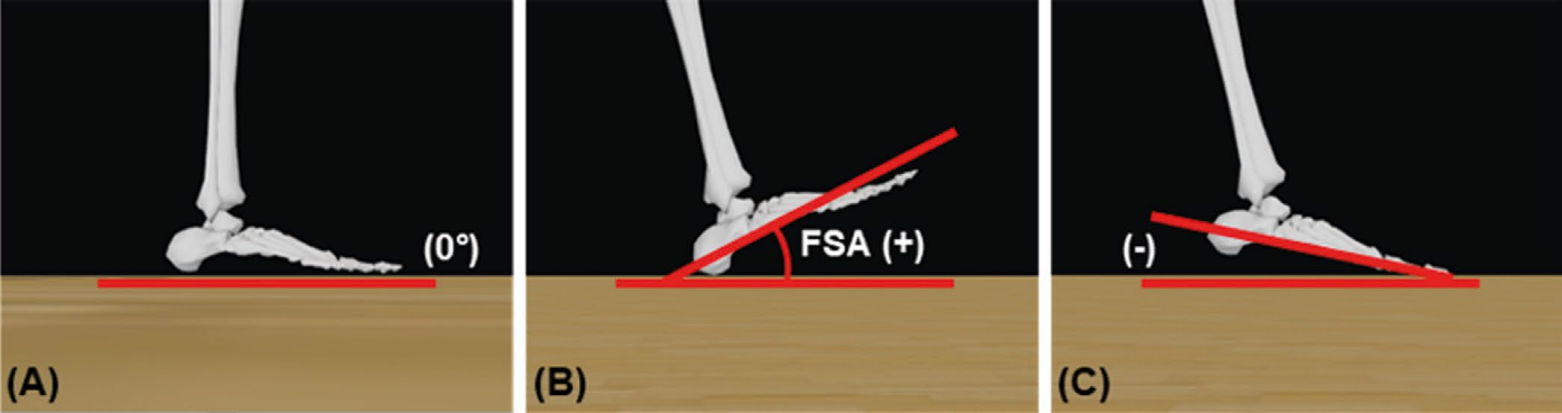
Definition of foot strike angle (FSA) in the sagittal plane: (A) an angle of 0° between the foot and the ground; (B) a heel contact landing with a positive angle; and (C) a forefoot landing with a negative angle. Images adapted from OpenSim software [36] for visualization purposes only; analyses were not conducted in OpenSim.

As reported by Altman and Davis [29], FSA was determined by measuring the angle between a straight line extending from the heel marker to the center marker on the dorsal aspect of the foot, specifically at the center of the 1st and 5th metatarsals, and the horizontal plane of the ground. This measurement was offset by the angle during standing, such that the standing angle represented 0° (Figure 2).

### 2.4 Statistical Analysis

Analysis of FSA, primary outcome variables (tibial peak bending moments, cumulative‐weighted tibial impulse per kilometer), and explanatory outcome variables (muscular component and JRF component) was conducted utilizing a one‐way repeated measures ANOVA using IBM SPSS Statistics (Version 26; IBM, Chicago, IL, USA) to assess the main effects of foot strike pattern during running. The assumption of sphericity was verified using Mauchly's test, and Greenhouse–Geisser corrections were used where appropriate. Effect sizes were measured using partial *η* ^2^ ($n_{p}^{2}$). Statistical significance was predetermined at *p <* 0.05. A post hoc test was conducted using the Bonferroni correction.

To enhance the visualization and interpretation of findings across the stance phase of running, statistical parametric mapping (SPM) was employed using the SPM1D package for MATLAB [38]. SPM facilitates the analysis of time‐series data, highlighting temporal patterns in tibial loading that discrete metrics may overlook. Consistent with previous studies [14, 39], this study used SPM to compare normalized time‐series data of tibial bending moments between foot strike patterns, excluding the initial and final 1% of the stance phase for physiologically meaningful comparisons. SPM analyses were included solely for visualization purposes, and no post‐hoc testing was conducted.

## 3 Results

### 3.1 Foot Strike Angle

**FIGURE 3:**
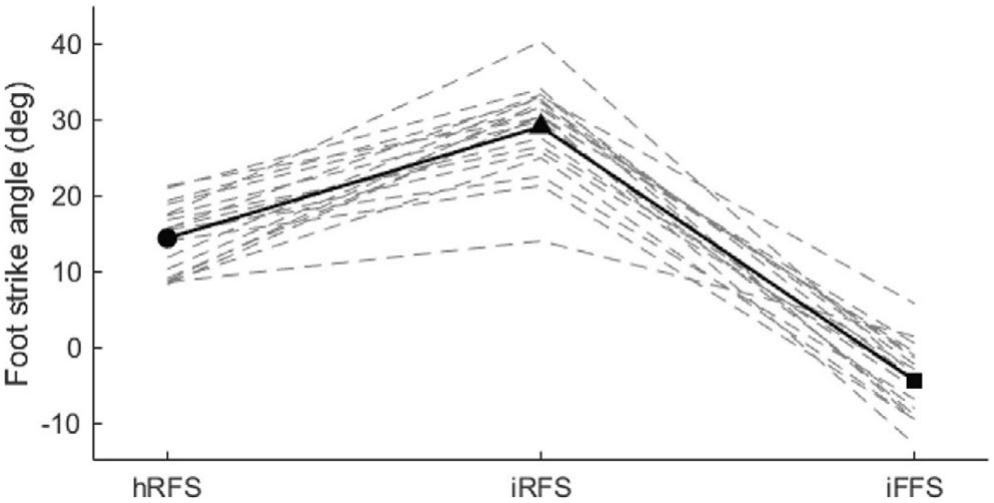
Mean foot strike angle for 19 participants across three conditions during five running trials per condition. Solid symbols represent the group mean. Dashed lines represent each individual's foot strike angle for each foot strike condition. hRFS, habitual rearfoot strike; iFFS, imposed forefoot strike; iRFS, imposed rearfoot strike.

There was a significant main effect of foot strike pattern on FSA (*p <* 0.001, *F* = 237.638). Post hoc analysis revealed significant differences in FSA among all conditions. Running with a habitual rearfoot strike (14.3° *±* 4.3°) was significantly different from both imposed conditions. Specifically, an imposed rearfoot strike produced a significantly greater FSA (29.0° *±* 5.6°, *p <* 0.001), whereas an imposed forefoot strike resulted in a significantly lower FSA (−4.4° *±* 4.6°, *p <* 0.001). Additionally, FSA values for the imposed rearfoot and forefoot striking patterns were significantly different from each other (*p <* 0.001) (Figure 3).

### 3.2 Primary Outcome Variables

#### 3.2.1 Tibial Peak Bending Moment

There was a significant main effect of foot strike pattern on tibial peak resultant bending moment (*p <* 0.001, *F* = 41.254) (Table 1). Post hoc analysis revealed that running with an imposed forefoot strike increased the tibial peak bending moment by 15.0% compared to a habitual rearfoot strike (*p <* 0.001) and by 35.7% compared to an imposed rearfoot strike (*p* = 0.001). Additionally, the tibial peak bending moment was significantly lower by 15.3% when running with an imposed rearfoot strike compared to a habitual rearfoot strike (*p <* 0.001).

**TABLE 1:** Primary and explanatory variables related to internal loading at the distal 1/3 of the tibia.

Variables	hRFS	iRFS	iFFS	*p*	$n_{p}^{2}$	Post‐hoc
*Primary*						
Resultant (Nm)	142.3 (34.9)	120.5 (24.3)	163.6 (34.1)	< 0.001	0.696	iFFS > iRFS, iFFS > hRFS, hRFS > iRFS
Cumulative per km (Nm^6.6^·s·km^−1^)^1/6.6^	229.1 (53.7)	189.9 (35.9)	265.7 (51.5)	< 0.001	0.728	iFFS > iRFS, iFFS > hRFS, hRFS > iRFS
*Explanatory*						
Muscular (Nm)	162.1 (34.4)	140.0 (24.6)	181.9 (37.8)	< 0.001	0.685	iFFS > iRFS, iFFS > hRFS, hRFS > iRFS
Reaction (Nm)	−46.9 (10.8)	−40.4 (9.7)	−49.6 (11.4)	< 0.001	0.650	iFFS > iRFS, iFFS > hRFS, hRFS > iRFS

*Note:* All post‐hoc results are significant at *p <* 0.05. Primary variables include tibial peak bending moment (resultant) and cumulative‐weighted tibial impulse per kilometer (cumulative per km), while explanatory variables include the muscular component of tibial peak bending moment (muscular) and the joint reaction force component of tibial peak bending moment (reaction). All values are presented as mean (SD).

Abbreviations: hRFS, habitual rearfoot strike; iFFS, imposed forefoot strike; iRFS, imposed rearfoot strike.

**FIGURE 4:**
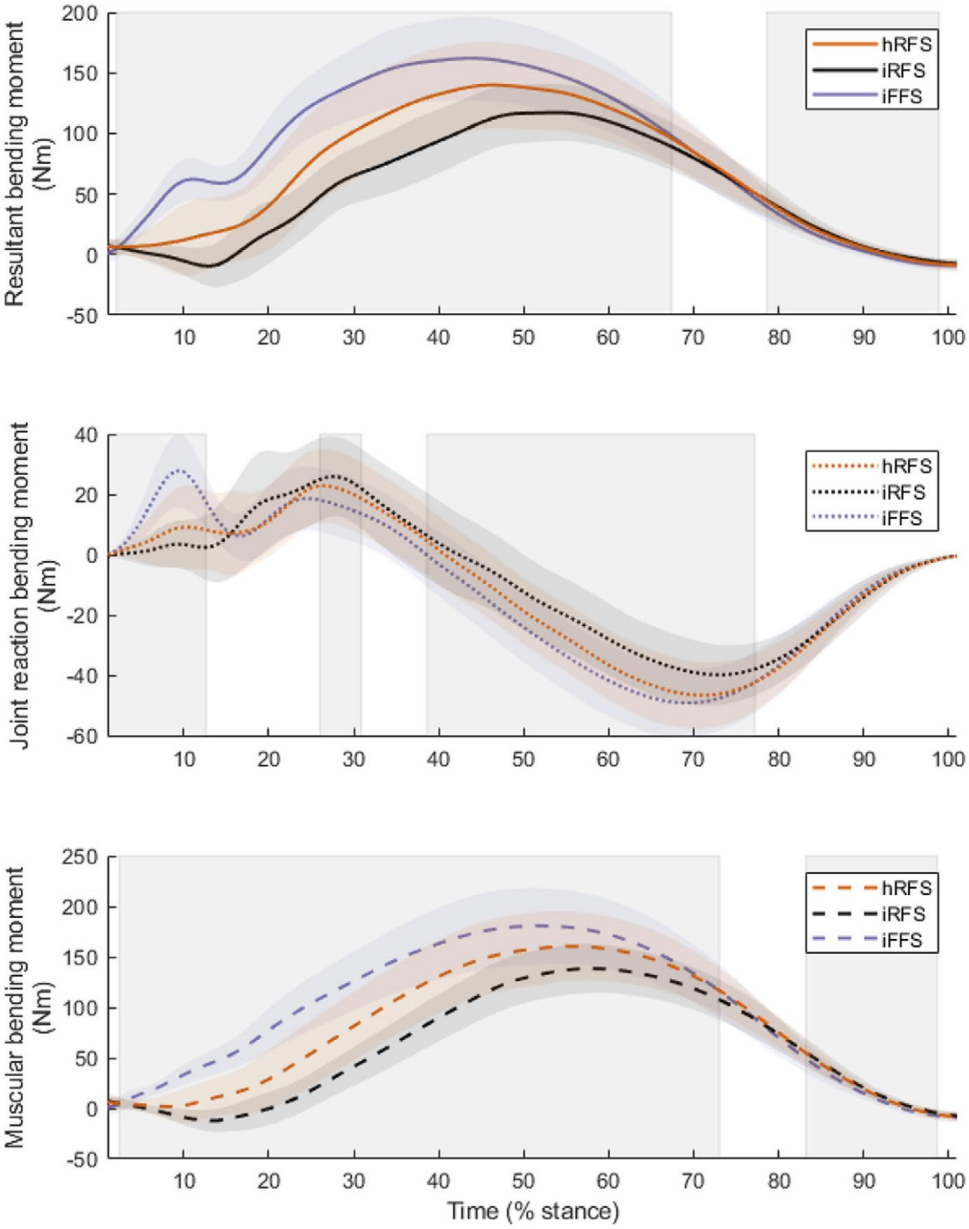
Mean and standard deviation time‐series of bending moments, including tibial bending moment (top), joint reaction force component of tibial bending moment (middle), and muscular component of tibial bending moment (bottom). Vertically shaded areas indicate regions where significant differences were observed between foot strike patterns (hRFS, habitual rearfoot strike; iFFS, imposed forefoot strike; iRFS, imposed rearfoot strike).

SPM analysis revealed significant main effects on the tibial bending moment between 2% and 67% of the stance phase and between 79% and 99% of the stance phase (both *p <* 0.001) (Figure 4).

#### 3.2.2 Cumulative‐Weighted Tibial Impulse per Kilometer

A significant main effect of foot strike pattern was observed for cumulative‐weighted tibial impulse per kilometer (*p <* 0.001, *F* = 48.086) (Table 1). Post hoc analysis revealed that running with an imposed forefoot strike resulted in a cumulative‐weighted tibial impulse per kilometer that was 15.9% greater than that observed with a habitual rearfoot strike (*p* = 0.001) and 39.9% greater than that observed with an imposed rearfoot strike (*p <* 0.001). Additionally, running with a habitual rearfoot strike produced a cumulative‐weighted tibial impulse per kilometer that was 20.6% greater than that of an imposed rearfoot strike (*p <* 0.001).

### 3.3 Explanatory Outcome Variables

#### 3.3.1 Muscular Component of Tibial Peak Bending Moment

A significant main effect of foot strike was observed for the muscular component (*p <* 0.001, *F* = 39.087). Post hoc analysis indicated that running with an imposed forefoot strike resulted in a significantly greater muscular component than running with a habitual rearfoot strike (*p* = 0.002) and an imposed rearfoot strike (*p <* 0.001). Furthermore, the muscular component was significantly lower when running with an imposed rearfoot strike compared to a habitual rearfoot strike (*p <* 0.001).

SPM analysis revealed significant main effects on the muscular component, with significant effects observed between 2% and 73% of the stance phase (*p <* 0.001) and between 83% and 99% of the stance phase (*p <* 0.001) (Figure 4).

#### 3.3.2 Joint Reaction Force Component of Tibial Peak Bending Moment

Foot strike pattern had a significant main effect on the JRF component (*p <* 0.001, *F* = 33.446). Post hoc analysis showed that running with an imposed forefoot strike resulted in a significantly greater JRF component than running with a habitual rearfoot strike (*p* = 0.043) and an imposed rearfoot strike (*p <* 0.001). The JRF component was significantly lower when running with an imposed rearfoot strike compared to running with a habitual rearfoot strike (*p <* 0.001).

SPM analysis also revealed significant main effects on the JRF component. Differences were observed between 1% and 13% of the stance phase (*p <* 0.001), between 26% and 31% (*p* = 0.017), and between 39% and 77% of the stance phase (*p <* 0.001) (Figure 4).

## 4 Discussion

This study evaluated the influence of foot strike manipulation on the loading of the distal 1/3 of the tibia in habitual rearfoot runners. The tibial bending moment in the sagittal plane caused the tibia to bend concave posteriorly through the majority of stance, leading to predominantly tensile stress in the anterior tibia and compressive stress in the posterior tibia, consistent with previous findings [12, 13, 14, 15, 40]. The tibial bending moment in the current study, when converted to equivalent units for comparison, was 12.9 ± 2.7 BW·HT, slightly higher than 7.77 ± 1.5 BW·HT reported by Phuah et al. [41] at a similar tibial location, 70% of the length from proximal to distal and when running at the same speed. This difference may stem from their inclusion of fewer muscles in the calculations.

Consistent with our hypothesis, greater bending moments were observed when running with a forefoot strike than a rearfoot strike. The time‐series analyses demonstrated increased tibial loading during running with an imposed forefoot strike throughout most of the stance phase, resulting also in higher peak values. The finding that transitioning from a habitual rearfoot strike to a more exaggerated rearfoot strike reduced tibial bending moments by 15.3%, whereas transitioning to a forefoot strike increased bending moments by 15.0% could be interpreted in terms of risk of tibial stress injury, where a greater magnitude of loading is understood to be one of the most important determinants of bone tissue damage [42]. However, in the present study, participants were not accustomed to running with these foot strikes, and it remains unclear whether such large magnitudes of difference would be observed if runners became accustomed to these foot strike patterns.

Bone remodels according to the stimuli it receives [6] such that the tissue may not be well adapted to withstand new patterns of loading, even if the magnitudes are lower. Transitioning to a new foot strike pattern involves substantial neuromuscular adjustments. For instance, Shih et al. [43] observed increased tibialis anterior activation during early stance when habitual rearfoot strikers transitioned to forefoot striking, reflecting the additional demands placed on the dorsi flexors during such transitions. In contrast, Yong et al. [44] found no significant differences in tibialis anterior and gastrocnemius activity during early stance between natural forefoot and rearfoot strikers, suggesting that habitual forefoot runners exhibit long‐term adaptations that reduce neuromuscular demands. These findings underscore the importance of gradual transitions to new foot strike patterns to allow sufficient neuromuscular and musculoskeletal adaptation.

**FIGURE 5:**
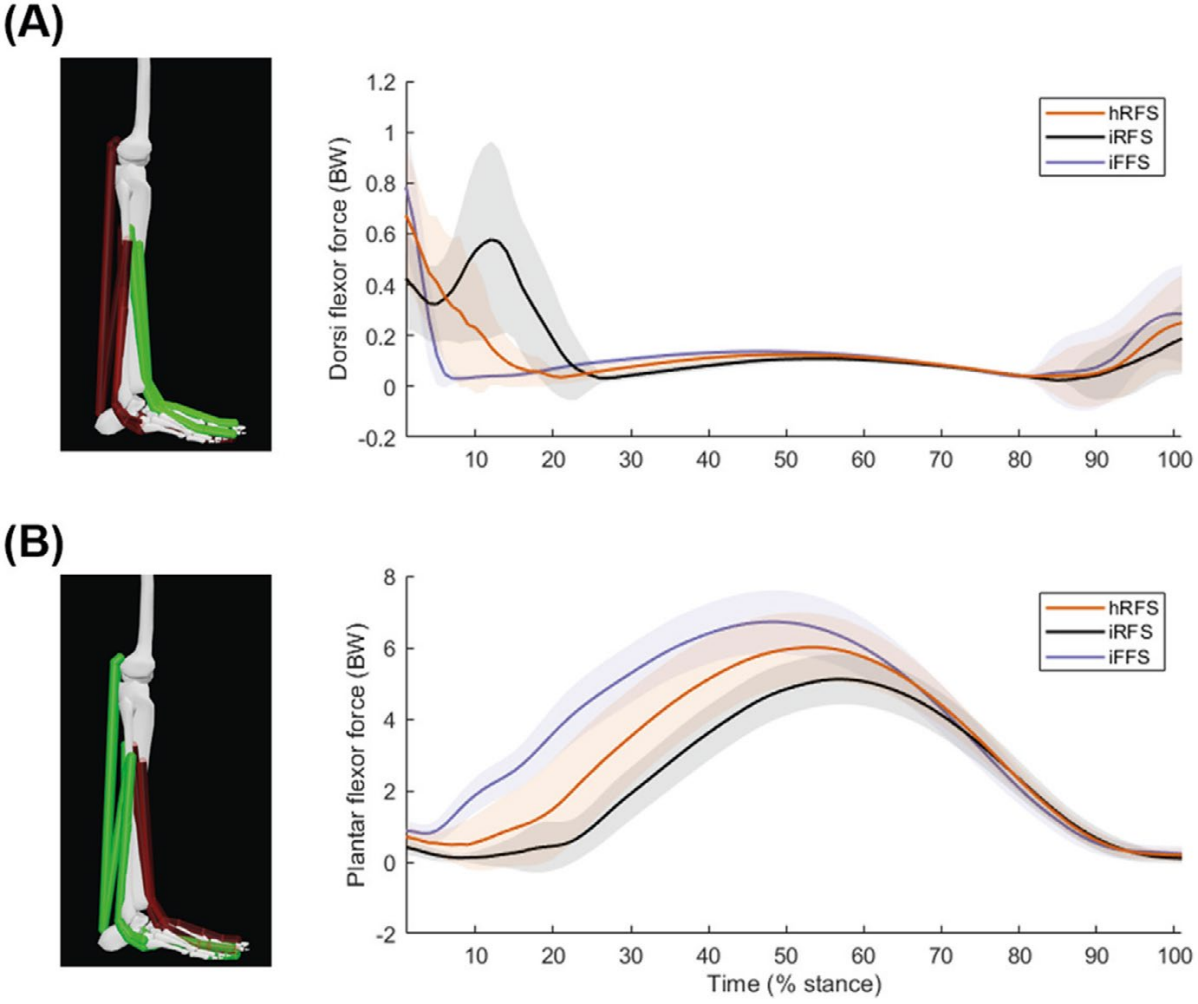
Mean and standard deviation time‐series of dorsi flexor (A) and plantar flexor (B) forces across the stance phase. Dorsi flexors include the tibialis anterior, extensor digitorum longus, and extensor hallucis longus. Plantar flexors include the lateral gastrocnemius, medial gastrocnemius, soleus, flexor digitorum longus, flexor hallucis longus, peroneus brevis, peroneus longus, and tibialis posterior. Shaded areas represent standard deviation. hRFS, habitual rearfoot strike; iFFS, imposed forefoot strike; iRFS, imposed rearfoot strike. Images adapted from OpenSim software [36] for visualization purposes only; analyses were not conducted in OpenSim.

The dorsi flexor and plantar flexor forces aid understanding of the modulating effects on tibial loading across different foot strike patterns (Figure 5). The dorsi flexors, including the tibialis anterior, extensor digitorum longus, and extensor hallucis longus, exhibit higher muscular forces during imposed rearfoot striking compared to both imposed forefoot striking and habitual rearfoot striking in the early stance phase. This elevated force production assists in decelerating the foot immediately after heel contact, as the dorsi flexors engage to manage the initial impact and control foot placement during rearfoot striking. Notably, imposed rearfoot striking is the only condition showing a loading pattern whereby the direction of the resultant bending moments is initially negative, before becoming positive (Figure 4). This is indicative of the posterior tibia initially undergoing tension before being compressed throughout the rest of stance, and vice versa at the anterior tibia. This could be attributed to increased activation of the dorsi flexors, which may exaggerate the mechanics of rearfoot striking. In contrast, this reverse loading pattern was not observed in habitual rearfoot and forefoot striking, where positive bending moments persisted throughout the stance phase. Although the implications of reverse loading for injury risk remain unclear, this pattern may influence bone remodeling. This concept aligns with Arndt et al. [45], who observed ‘cyclic unloading’ in metatarsal loading in nonfatigued states, though its direct relevance to tibial adaptation requires further research.

The plantar flexors produced higher muscular forces during imposed forefoot striking than rearfoot striking due to the greater work required at the ankle when running with a forefoot strike. These higher muscular forces are the main contributor to the increased resultant bending moments (Figure 4). In particular, this observation of greater plantar flexor forces when running with a forefoot strike corresponds with an earlier occurrence of peak force generation. The contribution of the plantar flexors is much greater than that of the dorsi flexors in early stance during forefoot striking, leading to initial posterior compression, which continues throughout the stance phase. Similarly, previous studies have reported that the ankle joint contact force, which is influenced by internal muscular forces spanning the tibia, was higher when transitioning from rearfoot to forefoot striking [22]. Understanding these distinct mechanical differences provides valuable insights for developing targeted training strategies to minimize injury risk. These insights suggest that transitioning from a habitual rearfoot strike to a forefoot strike may not be beneficial if seeking to reduce loading of the tibia. Therefore, footstrike transitioning should be conducted gradually to allow for physiological adaptations of the musculoskeletal structures in the lower extremities. However, interpretation of the results and translation into recommendations should be done cautiously, considering the limitations of the study.

One limitation of this study relates to the acute transition between foot strike patterns. Despite efforts to familiarize participants with forefoot striking, they were not accustomed to these imposed patterns. Additionally, the short 5‐m run‐up may have influenced results, potentially favoring certain foot strike types and not fully reflecting typical running conditions. The study also exclusively monitored the right foot, without considering potential differences between the dominant and nondominant leg. Jandovaet al. [46] demonstrated that laterality can affect running mechanics, suggesting that future studies should consider analyzing the dominant leg. Although verbal feedback and two sets of timing gates were used to encourage participants to maintain a consistent speed over the force plate, the setup did not directly confirm whether acceleration or braking occurred.

The study relied on computational modeling to estimate tibial loading. Although anthropometric parameters were gender‐specific, the model did not account for individual anatomical variations or tibial material properties, potentially affecting the precision of bending moment estimations. Another limitation concerns the cumulative‐weighted tibial impulse per kilometer variable, which was estimated from spatial–temporal data of a single overground trial and extrapolated to 1 km. This approach neglects physiological fatigue, which may alter biomechanical variables during longer runs. Furthermore, the estimate was based on a limited number of nonconsecutive foot strikes, which may limit the generalizability of the results.

Finally, reliance on static optimization to estimate muscle forces is a key limitation. This method depends on generalized net joint moments and does not account for individual‐specific muscle activation patterns or coordination strategies. By minimizing overall muscle activation, static optimization may underestimate co‐activation during ground contact. Previous research has shown this can lead to inaccurate muscle force predictions, particularly for muscles like the tibialis anterior, where model outputs often diverge from electromyography data [47]. This raises concerns about the validity of force estimates in dynamic, real‐world conditions.

## 5 Conclusion

In conclusion, this study demonstrates that running with an imposed nonhabitual forefoot strike results in higher internal tibial loading compared with rearfoot striking, primarily due to increased mechanical demands placed on the plantar flexors. This suggests that transitioning from a habitual rearfoot strike to a forefoot strike may not be advisable for runners aiming to reduce the magnitude of loading on the tibia.

## Author Contributions

S.H. conceived, planned, and designed the study, collected and analyzed data, and drafted, revised, and edited the manuscript. J.M. contributed to the study design, data collection, analysis, and manuscript revision. J.L. and S.H. assisted with recruitment, data collection, and manuscript revision. T.S. and D.F. contributed to methodological development and editing of the manuscript. H.R. supervised the study and contributed to data analysis, as well as the drafting and revision of the manuscript. All authors have read and approved the final version of the manuscript and agree with the author order.

## Conflicts of Interest

The authors declare no conflicts of interest.

## Data Availability

The data that support the findings of this study are available from the corresponding author upon reasonable request.

## References

[1] R. Van Gent , D. Siem , M. van Middelkoop , A. Van Os , S. Bierma‐Zeinstra , and B. Koes , “Incidence and Determinants of Lower Extremity Running Injuries in Long Distance Runners: A Systematic Review,” British Journal of Sports Medicine 41, no. 8 (2007): 469–480, 10.1136/bjsm.2006.033548. 17473005 PMC2465455

[2] G. A. Robertson and A. M. Wood , “Lower Limb Stress Fractures in Sport: Optimising Their Management and Outcome,” World Journal of Orthopedics 8, no. 3 (2017): 242, 10.5312/wjo.v8.i3.242. 28361017 PMC5359760

[3] J. Iwamoto and T. Takeda , “Stress Fractures in Athletes: Review of 196 Cases,” Journal of Orthopaedic Science 8, no. 3 (2003): 273–278, 10.1007/s10776-002-0632-5. 12768465

[4] D. Burr , M. R. Forwood , D. P. Fyhrie , R. B. Martin , M. B. Schaffler , and C. H. Turner , “Bone Microdamage and Skeletal Fragility in Osteoporotic and Stress Fractures,” Journal of Bone and Mineral Research 12, no. 1 (1997): 6–15, 10.1359/jbmr.1997.12.1.6. 9240720

[5] S. J. Warden , I. S. Davis , and M. Fredericson , “Management and Prevention of Bone Stress Injuries in Long‐ Distance Runners,” Journal of Orthopaedic & Sports Physical Therapy 44, no. 10 (2014): 749–765, 10.2519/jospt.2014.5334. 25103133

[6] H. M. Frost , “Wolff's Law and Bone's Structural Adaptations to Mechanical Usage: An Overview for Clinicians,” Angle Orthodontist 64, no. 3 (1994): 175–188. 8060014 10.1043/0003-3219(1994)064<0175:WLABSA>2.0.CO;2

[7] D. Burr , C. Turner , P. Naick , M. Forwood , and R. Pidaparti , “Does Bone Microdamage Affect the Normal Mechanical Properties of Bone,” Transactions of the Orthopaedic Research Society 20, no. 2 (1995): 127.

[8] L. Lanyon , W. Hampson , A. Goodship , and J. Shah , “Bone Deformation Recorded In Vivo From Strain Gauges Attached to the Human Tibial Shaft,” Acta Orthopaedica Scandinavica 46, no. 2 (1975): 256–268. 1146518 10.3109/17453677508989216

[9] D. Burr , C. Milgrom , D. Fyhrie , et al., “In Vivo Measurement of Human Tibial Strains During Vigorous Activity,” Bone 18, no. 5 (1996): 405–410, 10.1016/8756-3282(96)00028-2. 8739897

[10] C. Milgrom , A. Finestone , Y. Levi , et al., “Do High Impact Exercises Produce Higher Tibial Strains Than Running?,” British Journal of Sports Medicine 34, no. 3 (2000): 195–199, 10.1136/bjsm.34.3.195. 10854019 PMC1763261

[11] C. Milgrom , D. R. Radeva‐Petrova , A. Finestone , et al., “The Effect of Muscle Fatigue on In Vivo Tibial Strains,” Journal of Biomechanics 40, no. 4 (2007): 845–850, 10.1016/j.jbiomech.2006.03.006. 16682046

[12] T. R. Derrick , W. B. Edwards , R. E. Fellin , and J. F. Seay , “An Integrative Modeling Approach for the Efficient Estimation of Cross Sectional Tibial Stresses During Locomotion,” Journal of Biomechanics 49, no. 3 (2016): 429–435, 10.1016/j.jbiomech.2016.01.003. 26803338

[13] S. A. Meardon and T. R. Derrick , “Effect of Step Width Manipulation on Tibial Stress During Running,” Journal of Biomechanics 47, no. 11 (2014): 2738–2744, 10.1016/j.jbiomech.2014.04.047. 24935171

[14] H. Rice , G. Weir , M. B. Trudeau , S. A. Meardon , T. R. Derrick , and J. Hamill , “Estimating Tibial Stress Throughout the Duration of a Treadmill Run,” Medicine & Science in Sports & Exercise 51, no. 11 (2019): 2257–2264, 10.1249/MSS.0000000000002039. 31634292

[15] H. Rice , M. Kurz , P. Mai , et al., “Speed and Surface Steepness Affect Internal Tibial Loading During Running,” Journal of Sport and Health Science 13, no. 1 (2024): 118–124, 10.1016/j.jshs.2023.03.004. 36931595 PMC10818105

[16] W. B. Edwards , “Modeling Overuse Injuries in Sport as a Mechanical Fatigue Phenomenon,” Exercise and Sport Sciences Reviews 46, no. 4 (2018): 224–231, 10.1249/JES.0000000000000163. 30001271

[17] C. R. Firminger , M. J. Asmussen , S. Cigoja , J. R. Fletcher , B. M. Nigg , and W. B. Edwards , “Cumulative Metrics of Tendon Load and Damage Vary Discordantly With Running Speed,” Medicine & Science in Sports & Exercise 52, no. 7 (2020): 1549–1556, 10.1249/MSS.0000000000002287. 31985576

[18] H. Rice , P. Mai , M. Sanno , and S. Willwacher , “Tibial Loading and Damage Accumulation in Recreational and Competitive Male Runners During a Demanding 10 km Run,” European Journal of Sport Science 24, no. 1 (2024): 79–87, 10.1002/ejsc.12040.

[19] A. I. Daoud , G. J. Geissler , F. Wang , J. Saretsky , Y. A. Daoud , and D. E. Lieberman , “Foot Strike and Injury Rates in Endurance Runners: A Retrospective Study,” Medicine & Science in Sports & Exercise 44, no. 7 (2012): 1325–1334, 10.1249/mss.0b013e3182465115. 22217561

[20] E. R. Boyer , B. D. Rooney , and T. R. Derrick , “Rearfoot and Midfoot or Forefoot Impacts in Habitually Shod Runners,” Medicine and Science in Sports and Exercise 46, no. 7 (2014): 1384–1391, 10.1249/mss.0000000000000234. 24300124

[21] A. A. Zadpoor and A. A. Nikooyan , “The Relationship Between Lower‐Extremity Stress Fractures and the Ground Reaction Force: A Systematic Review,” Clinical Biomechanics 26, no. 1 (2011): 23–28, 10.1016/j.clinbiomech.2010.08.005. 20846765

[22] T. L. Chen , W. W. An , Z. Chan , I. P. H. Au , Z. Zhang , and R. Cheung , “Immediate Effects of Modified Landing Pattern on a Probabilistic Tibial Stress Fracture Model in Runners,” Clinical Biomechanics 33 (2016): 49–54, 10.1016/j.clinbiomech.2016.02.013. 26945721

[23] S. Sasimontonkul , B. K. Bay , and M. J. Pavol , “Bone Contact Forces on the Distal Tibia During the Stance Phase of Running,” Journal of Biomechanics 40, no. 15 (2007): 3503–3509, 10.1016/j.jbiomech.2007.05.024. 17662295

[24] E. S. Matijevich , L. M. Branscombe , L. R. Scott , and K. E. Zelik , “Ground Reaction Force Metrics Are Not Strongly Correlated With Tibial Bone Load When Running Across Speeds and Slopes: Implications for Science, Sport and Wearable Tech,” PLoS One 14, no. 1 (2019): e0210000, 10.1371/journal.pone.0210000. 30653510 PMC6336327

[25] Y. Huang , H. Xia , G. Chen , S. Cheng , R. T. Cheung , and P. B. Shull , “Foot Strike Pattern, Step Rate, and Trunk Posture Combined Gait Modifications to Reduce Impact Loading During Running,” Journal of Biomechanics 86 (2019): 102–109, 10.1016/j.jbiomech.2019.01.058. 30792072

[26] J. R. Yong , A. Silder , K. L. Montgomery , M. Fredericson , and S. L. Delp , “Acute Changes in Foot Strike Pattern and Cadence Affect Running Parameters Associated With Tibial Stress Fractures,” Journal of Biomechanics 76 (2018): 1–7, 10.1016/j.jbiomech.2018.05.017. 29866518 PMC6203338

[27] M. Keast , J. Bonacci , and A. Fox , “Acute Effects of Gait Interventions on Tibial Loads During Running: A Systematic Review and Meta‐Analysis,” Sports Medicine 52, no. 10 (2022): 2483–2509, 10.1007/s40279-022-01703-1. 35708887 PMC9474464

[28] S. M. Stearne , J. A. Alderson , B. A. Green , C. J. Donnelly , and J. Rubenson , “Joint Kinetics in Rearfoot Versus Forefoot Running: Implications of Switching Technique,” Medicine & Science in Sports & Exercise 46, no. 8 (2014): 1578–1587, 10.1249/mss.0000000000000254. 24500531

[29] A. R. Altman and I. S. Davis , “A Kinematic Method for Footstrike Pattern Detection in Barefoot and Shod Runners,” Gait & Posture 35, no. 2 (2012): 298–300, 10.1016/j.gaitpost.2011.09.104. 22075193 PMC3278526

[30] G. Shan and C. Bohn , “Anthropometrical Data and Coefficients of Regression Related to Gender and Race,” Applied Ergonomics 34, no. 4 (2003): 327–337, 10.1016/s0003-6870(03)00040-1. 12880743

[31] A. Erdemir , S. McLean , W. Herzog , and A. J. van den Bogert , “Model‐Based Estimation of Muscle Forces Exerted During Movements,” Clinical Biomechanics 22, no. 2 (2007): 131–154, 10.1016/j.clinbiomech.2006.09.005. 17070969

[32] M. Baggaley , T. R. Derrick , and B. W. Edwards , “Sensitivity of Internal Tibial Forces and Moments to Static Optimization Moment Constraints at the Subtalar and Ankle Joints,” Journal of Biomechanical Engineering 145, no. 1 (2023): 011008, 10.1115/1.4055036. 35864788

[33] E. M. Arnold , S. R. Ward , R. L. Lieber , and S. L. Delp , “A Model of the Lower Limb for Analysis of Human Movement,” Annals of Biomedical Engineering 38 (2010): 269–279, 10.1007/s10439-009-9852-5. 19957039 PMC2903973

[34] S. R. Hamner , A. Seth , and S. L. Delp , “Muscle Contributions to Propulsion and Support During Running,” Journal of Biomechanics 43, no. 14 (2010): 2709–2716, 10.1016/j.jbiomech.2010.06.025. 20691972 PMC2973845

[35] C. Milgrom , M. Gildadi , A. Simkin , et al., “The Area Moment of Inertia of the Tibia: A Risk Factor for Stress Fractures,” Journal of Biomechanics 22, no. 11–12 (1989): 1243–1248, 10.1016/0021-9290(89)90226-1. 2625424

[36] S. L. Delp , F. C. Anderson , A. S. Arnold , et al., “OpenSim: Open‐Source Software to Create and Analyze Dynamic Simulations of Movement,” IEEE Transactions on Biomedical Engineering 54, no. 11 (2007): 1940–1950, 10.1109/tbme.2007.901024. 18018689

[37] C. R. Firminger and W. B. Edwards , “The Influence of Minimalist Footwear and Stride Length Reduction on Lower‐Extremity Running Mechanics and Cumulative Loading,” Journal of Science and Medicine in Sport 19, no. 12 (2016): 975–979, 10.1016/j.jsams.2016.03.003. 27107980

[38] T. C. Pataky , M. A. Robinson , and J. Vanrenterghem , “Region‐of‐Interest Analyses of One‐Dimensional Biomechanical Trajectories: Bridging 0D and 1D Theory, Augmenting Statistical Power,” PeerJ 4 (2016): e2652, 10.7717/peerj.2652. 27833816 PMC5101620

[39] H. Rice , M. Kenny , M. Ellison , et al., “Tibial Stress During Running Following a Repeated Calf‐Raise Protocol,” Scandinavian Journal of Medicine & Science in Sports 30, no. 12 (2020): 2382–2389, 10.1111/sms.13794. 32757284

[40] P. F. Yang , M. Sanno , B. Ganse , et al., “Torsion and Antero‐Posterior Bending in the In Vivo Human Tibia Loading Regimes During Walking and Running,” PLoS One 9, no. 4 (2014): e94525, 10.1371/journal.pone.0094525. 24732724 PMC3986088

[41] A. H. Phuah , A. G. Schache , K. M. Crossley , T. V. Wrigley , and M. W. Creaby , “Sagittal Plane Bending Moments Acting on the Lower Leg During Running,” Gait & Posture 31, no. 2 (2010): 218–222. 19926481 10.1016/j.gaitpost.2009.10.009

[42] D. Carter and W. Caler , “A Cumulative Damage Model for Bone Fracture,” Journal of Orthopaedic Research 3, no. 1 (1985): 84–90, 10.1002/jor.1100030110. 3981298

[43] Y. Shih , K. L. Lin , and T. Y. Shiang , “Is the Foot Striking Pattern More Important Than Barefoot or Shod Conditions in Running?,” Gait & Posture 38, no. 3 (2013): 490–494, 10.1016/j.gaitpost.2013.01.030. 23507028

[44] J. R. Yong , A. Silder , and S. L. Delp , “Differences in Muscle Activity Between Natural Forefoot and Rearfoot Strikers During Running,” Journal of Biomechanics 47, no. 15 (2014): 3593–3597, 10.1016/j.jbiomech.2014.10.015. 25458201 PMC4301610

[45] A. Arndt , I. Ekenman , P. Westblad , and A. Lundberg , “Effects of Fatigue and Load Variation on Metatarsal Deformation Measured In Vivo During Barefoot Walking,” Journal of Biomechanics 35, no. 5 (2002): 621–628, 10.1016/s0021-9290(01)00241-x. 11955501

[46] S. Jandova´ , P. Volf , and F. Vaverka , “The Influence of Minimalist and Conventional Sports Shoes and Lower Limbs Dominance on Running Gait,” Acta of Bioengineering and Biomechanics 20, no. 3 (2018): 3–9, 10.5277/ABB-01120-2018-02. 30520437

[47] M. Baggaley , T. R. Derrick , G. Vernillo , G. Y. Millet , and W. B. Edwards , “Internal Tibial Forces and Moments During Graded Running,” Journal of Biomechanical Engineering 144, no. 1 (2022): 011009, 10.1115/1.4051924. 34318310

